# Demonstration of an E-mailed Worksite Nutrition Intervention Program

**Published:** 2004-09-15

**Authors:** Gladys Block, Torin Block, Patricia Wakimoto, Clifford H. Block

**Affiliations:** School of Public Health, University of California; Block Dietary Data Systems, Berkeley, Calif; School of Public Health, University of California, Berkeley, Calif; Block Dietary Data Systems, Berkeley, Calif

## Abstract

**Introduction:**

Dietary fat and low fruit and vegetable intake are linked to many chronic diseases, and U.S. population intake does not meet recommendations. Interventions are needed that incorporate effective behavior-change principles and that can be delivered inexpensively to large segments of the population.

**Methods:**

Employees at a corporate worksite were invited to participate in a program, delivered entirely by e-mail, to reduce dietary fat and increase fruit and vegetable intake. Behavior-change principles underlying the intervention included tailoring to the participant's dietary lifestyle, baseline assessment and feedback about dietary intake, family participation, and goal setting. Assessment, tailoring, and delivery was fully automated. The program was delivered weekly to participants' e-mail inboxes for 12 weeks. Each e-mail included information on nutrition or on the relationship between diet and health, dietary tips tailored to the individual, and small goals to try for the next week. In this nonrandomized pilot study, we assessed technical feasibility, acceptability to employees, improvement in Stage of Change, increase in fruit and vegetable consumption, and decrease in fat intake.

**Results:**

Approximately one third (n = 84) of employees who were offered the 12-week program signed up for it, and satisfaction was high. There was significant improvement in Stage of Change: 74% of those not already at the top had forward movement (*P *<.001). In addition, results suggest significant increase in fruit and vegetable consumption (0.73 times/day, *P *<.001) and significant decrease in intake of fat sources (-0.39 times/day,* P* < .001).

**Conclusion:**

This inexpensive program is feasible and appears to be effective. A randomized controlled trial is needed.

## Introduction

Diets high in fat and low in fruits and vegetables have been associated with numerous health outcomes, including cardiovascular disease, cancer, diabetes, and obesity (1). Unfortunately, despite nutrition education and health campaigns, more than 80% of Americans do not meet dietary recommendations for these factors (2,3).

Large-scale programs such as the 5 A Day for Better Health program and campaigns aimed at reducing fat intake have increased awareness in many Americans, but changes in actual dietary habits are small (4). In part, this may be because such campaigns cannot incorporate some important behavior-change principles, such as personally relevant motivators, goal setting, and tailoring to the individual's characteristics.

In-person counseling can and does use those principles, and there is ample evidence that it is possible to improve dietary behaviors through intensive counseling and intervention (5,6). Successful worksite interventions have involved multiyear integrated programs, often with individual, face-to-face counseling (7,8). Intensive interventions such as these, however, are not feasible for most public health settings or primary care practices, and costs limit their usefulness in most worksite settings. Effective methods are needed that can be delivered broadly yet inexpensively.

The purpose of the e-mailed Worksite Internet Nutrition (WIN) program is to fill this gap by applying effective behavior-change principles on a large scale through technology. Computer technology permits the application of behavioral principles such as tailoring of messages to participant characteristics. E-mail technology permits the delivery of such effective programs directly to the participant. The WIN program was developed to deliver effective, science-based interventions and deliver them broadly and inexpensively.

The California Cancer Research Program of the California Department of Health Services (DHS) provided funding for the WIN program development and for the pilot study described here to test the technical feasibility and acceptability of the program to recipients in a corporate worksite. Although we tested the program in a worksite setting, it could be used in any setting with e-mail access. In addition to feasibility and acceptability results, we also provide data on evidence of effectiveness in promoting dietary behavior change and in Stage of Readiness for Change.

## Methods

The WIN program is a 12-week nutrition intervention delivered entirely by e-mail. The goal of WIN is to move people toward a more healthful diet with respect to dietary fat, fruits, and vegetables. The program was reviewed by the California DHS Institutional Review Board.

### Principles of the WIN program

The WIN program is based on principles of effective health education and behavior change. The principles incorporated into WIN are described below, and the program components through which they were implemented are shown in Table 1. Underlying these principles is a model of behavior change that includes the following characteristics: One, the individual *first needs to engage* with information that may be useful to him or her. An initial introduction to all members of the worksite was used to generate "community" interest in exploring this health intervention. The promise of a personalized assessment of dietary habits provided a significant initial incentive. Two, once engaged, the individual needs to be *moved to undertake some initial action*, even if it is a modest "small step," to begin to change from a passive recipient of information to a practitioner of new behaviors. The simple choice of pursuing either a fat-reducing program or a fruit- and vegetable-enhancing program provided an easy first step. Subsequently, very simple, lifestyle-relevant food practices made for an easy next step to initial behavioral changes. Three, once those first new behaviors are experienced as achievable, *enhanced self-efficacy can facilitate the acquisition of a whole cluster of relevant new behaviors*. Each week, reinforcement was provided in addition to a choice of new "small steps." Four, this process needs to continue long enough to *establish this new complex of behaviors as a habitual part of a person's daily routine*. The regularity and ease of responding to an e-mail–delivered message made it very easy to continue in the program over many weeks. The novelty of new information and new behavioral guidance each week also helped sustain participation. Finally, a steadily increasing understanding of the health impacts of diet raised the salience of this aspect of the individual's lifestyle. The intent was to increase the sustainability of more healthy behaviors, both those suggested by WIN and others.

The principles implemented in the WIN program are discussed below.

#### 1. Relevance to the learner

Research has shown that many people overestimate their fruit and vegetable intake (9) and think their own dietary intake needs no improvement (10). Following Weinstein's Precaution Adoption Process model (11), baseline evidence of personal risk behavior is an essential precursor to successful behavior change. A baseline dietary screening questionnaire was critical in implementing this principle. The questionnaire results provided participants with immediate estimates of their fat, fruit and vegetable, and dietary fiber intake in relation to recommended levels.

#### 2. Tailoring to the individual

To be acted upon, behavioral recommendations must be not only perceived to be needed but also must be feasible in the context of the individual's lifestyle (5,12). Through the baseline questionnaire, individuals were placed into one of seven "lifestyle paths" (Table 2), which reflected constraining lifestyle characteristics and determined the types of advice and goals that would most benefit the participant. Such patient-centered counseling provides support to participants while respecting their limitations (12). Program elements contributing to this principle are shown in Table 1.

#### 3. Flexibility and individual choice

The ability to make individual choices enhances participation and attention (13). Participants could choose one of two dietary emphases: reducing their fat intake or increasing their fruit and vegetable intake. Throughout the program, several behavioral tips and several goals were presented each week from which the participant could choose for his or her actions during the following week (Table 1).

#### 4. Skill facilitation

Skills in making healthier food choices and behaviors were enhanced through *tips *on easy ways to increase fruit and vegetable intake or decrease fat intake; *nutrition information* (e.g., "What is a serving?"); *links* to sites providing recipes or health information; and the *sharing* of strategies and ideas with coworkers via an online bulletin board.

#### 5. Commitment and goal setting

Extensive research indicates that goal setting is an important component of successful behavior change (14). Each week, the program presented four small, easily achievable goals that would move participants in the desired direction. Examples of such goals are shown in Table 2. One such goal might have been "I will put a bowl of fruit on the kitchen table this week."

#### 6. Reminders and reinforcement

Reminders keep the topic salient, and reinforcement helps to increase self efficacy. Reminders and reinforcement were provided to participants by the weekly messages, goal-setting opportunities, and opportunity to comment on their success. In addition, family members were encouraged to participate in the program, providing additional reminders and support.

#### 7. Multiple strategies and channels

To achieve behavior change, learners typically must hear messages from different people, in different contexts and repeatedly (15). We accomplished this goal in the following ways:

- Family participation and social support. Family members could also sign up for WIN, and were encouraged to do so, creating a supportive environment for the participant.
- Bulletin board to facilitate development of social networks and social support for behavior change.
- *Did you know?* and *Health Notes *information (described below) to stimulate discussion and raise interest and awareness.
- Electronic links to other sites, such as the National Cancer Institute, National Heart, Lung and Blood Institute, and the American Dietetic Association.

### Recruitment and data collection

**Figure 1:**
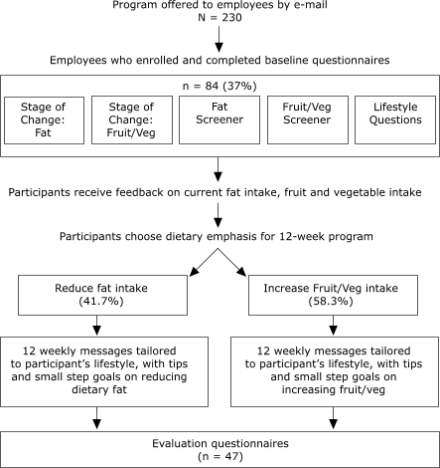
Flowchart illustrating how individuals took part in the Worksite Nutrition Intervention Program, Northern California, 2000.

WIN was piloted at a corporate worksite employing 230 individuals (Figure 1). Employees were sent an initial e-mail from the participating company, indicating that the program was authorized by the company, that participation was voluntary, and that all further interactions between employees and the WIN program would be independent of the company to guarantee confidentiality. Each employee was sent a baseline questionnaire and an informed consent statement, and an employee was considered enrolled in the program upon submission of both forms. For the subsequent 12 weeks, participants received a weekly automated e-mail directly from the WIN program.

### Measures

Dietary fat, fiber, and fruit and vegetable intake were assessed in the initial e-mail using the Block screening questionnaires (16,17). A separate lifestyle questionnaire asked for demographic information and information needed for individual tailoring, such as whether the respondent did most of the cooking, whether many meals were eaten out, and whether there were children at home.

Stage of Readiness for Change was assessed at baseline and at the end of the 12-week program. It was categorized in three stages, corresponding to Precontemplation, Contemplation/Preparation, and Action/Maintenance (18). Self-efficacy (i.e., confidence in the ability to make changes) was assessed at baseline, separately for increasing fruits and vegetables and decreasing fat. After the 12-week program, questions addressing Stage of Change were repeated, and we also sent participants questions on program satisfaction and a follow-up questionnaire on diet.

### Structure of e-mails to participants

Each message contained the following components:

- *"Did you know…?"* These were brief, interesting facts, designed to catch the attention of and promote discussion among recipients. Most included a table or figure and a link to Internet sources of further information.
- *Health Notes*. These were more extensive sections containing scientific information on nutrition or on the link between diet and health. For example, one *Health Notes* summarized evidence for the role of fruits and vegetables in reducing cancer risk. Others focused on the role of folic acid, the Food Guide Pyramid, "What is a serving?," heart disease, and other topics.
- *Tips and Ideas*. Each week, four new tips and suggestions focused on easily achievable actions. They were tailored to the participant in two ways: in their chosen dietary emphasis (i.e., reduce fat or increase fruits and vegetables) and in their lifestyle path. While some tips applied to all persons with the particular dietary emphasis (e.g., "Put a bowl of fruit on the kitchen table"), others applied to both dietary emphasis and lifestyle path (e.g., "You can get your vegetables even if you eat out a lot by choosing salads, baked potatoes, and so forth"). Table 2 gives examples of the types of tailored tips and ideas.
- *Goals for next week*. Each week, four new goals were suggested, usually related to the "Tips and Ideas" provided that week. Participants were asked to choose one or two to try during the following week. Goals were tailored to dietary emphasis and lifestyle path. (Examples are shown in Table 2.)

Messages were developed by registered dietitians or by one of the authors (GB, PW). For this 12-week program, 168 sets of messages were developed (12 weeks x 2 dietary emphases x 7 lifestyle paths). The program was completely automated; computer programming determined the lifestyle characteristics for each individual and delivered weekly tailored messages.

### Statistical methods

Linear regression and correlation techniques were used for continuous data, and classification and chi-square evaluation for categorical data. Differences between respondents and nonrespondents to the evaluation questionnaire were examined, and variables that differed were evaluated for confounding. To evaluate the effectiveness of the program, analysis of covariance was used, with change score as the dependent variable and baseline level as a covariate.

Raw scores ranged from 0–5 for each of the seven fruit and vegetable items and from 0–4 for each of the 17 dietary fat items. Scores represented categories of frequency of consumption, from "rarely/never" to "every day" for fat items or to "2+/day" for fruit and vegetable items. We then converted the responses to times per day. The follow-up questionnaire on diet presented participants with the same food items and their initial responses on fat, fiber, and fruits and vegetables, and assessed differences in frequency consumed (i.e., more, less, or the same frequency of each item). Changes in responses were summed over the 17 fat items and the seven fruit and vegetable items.

Additionally, we created a separate Change in Stage-of-Change score for fat and fruits and vegetables, in which 0 indicated no change, 1 indicated progression by one step (e.g., from Precontemplation to Contemplation/Preparation), and 2 indicated progression by two steps (i.e., from Precontemplation to Action/Maintenance).

To avoid the potential self-selection bias inherent in the 56% response rate to the evaluation questionnaire, persons who did not respond to the follow-up questionnaire were assigned a follow-up score identical to their baseline score. This conservative approach assumes that those who did not respond had no improvement. To avoid an artificially low variance for such nonrespondents, change in dietary intake was calculated after adding a random number to their imputed follow-up score. The random number, with mean equal to zero and standard deviation equal to the standard deviation of the participants who did return a follow-up score, could be either negative or positive. The resulting imputed follow-up score thus had a mean equal to the baseline score for these nonrespondents, but had a variance similar to the variance among respondents.

In addition to the self-reports obtained in the final evaluation questionnaire, the program automatically captured the number of times the participant interacted with the program by choosing a goal or using the online bulletin board. This variable was not biased by self-report and was used in analyses of internal consistency and dose-response.

## Results

Of the 84 persons participating in the 12-week program, age ranged from 21–63, and 73% were female. Forty-one percent said they had children at home, 72% said they do most of the food preparation, and 54% said they were budget-conscious when purchasing food. At baseline, approximately 50% were in the Precontemplation or Contemplation/Preparation stage of change in fat intake, and 46% were in those stages of change in fruit and vegetable intake.

Forty-seven participants (56%) completed the evaluation questionnaire at the end of the 12-week program. Nonrespondents to the evaluation questionnaire were more likely to have been in the Action/Maintenance Stage at baseline, but this difference was not statistically significant. Respondents and nonrespondents did not differ significantly in baseline fat score (2.1, respondents vs 2.3, nonrespondents), fruit and vegetable score (2.6, respondents vs 3.1, nonrespondents), lifestyle path, dietary emphasis, or confidence in ability to make changes. Men were more likely to complete the evaluation questionnaire than women were (76% of men vs 49% of women, *P* = .03), and mean age among evaluation respondents was slightly older compared to nonrespondents (*P* = .06).

### Feasibility and satisfaction

A key goal of the pilot project was to determine the feasibility of developing and delivering an extensive intervention via e-mail. Feasibility was clearly established. Only 6% of respondents reported any technical difficulties (Table 3). More than 93% of respondents found the nutrition tips and goals helpful. Approximately 83% would recommend the program to others. The majority found the amount of time the program took to be about right, but a substantial number (25%) would have preferred it to be shorter. Discussion about improving dietary habits was stimulated: almost half of respondents talked about it at home and one third talked about it with someone at work. These favorable results would be even higher but for the inclusion of six evaluation respondents who reported at baseline that they already ate a healthful diet and stated at follow-up that they therefore had not tried to change their diet.

We also examined the factors influencing whether or not respondents would recommend the program to others. Among the 50% of participants who said they were budget-conscious, 100% of them would recommend the program, while the proportion was lower (77%) among the non-budget–conscious. Similarly, among the 50% of the participants who at baseline did not claim to already have a healthful diet, 100% would recommend the program, while 76% of the already "health-conscious/healthful diet" would do so. Perhaps consistent with the health-consciousness observation, 100% of the male respondents would recommend the program, and 83% of the women would do so.

### Improvement in Stage of Change

Forward movement in Stage of Change was notable and statistically significant (Table 4). This was true even when nonrespondents to the evaluation questionnaire were included and assigned a change score of zero. Among evaluation respondents who were not already in "Action" at baseline (and therefore had room for forward movement), 65% had forward movement in Stage of Change for fat, and 74% had forward movement in improving fruit and vegetable intake.

We examined the data for internal consistency. The number of weeks that each person interacted with the program was captured by the program rather than self-report. Thus, all initial participants could be included (n = 84, with change equal to zero for nonrespondents), and the variable "number of weeks interacted" was not subject to reporting bias among those who responded to the evaluation questionnaire. In multiple linear regression, the number of weeks the participants interacted with the program was significantly related to change in Stage of Change for fruits and vegetables (*P* = .03) and change in Stage of Change for fat (*P* = .04) (data not shown). This was true even though change had been set to zero for persons not responding to the evaluation questionnaire.

### Dietary changes

Among respondents to the evaluation questionnaire, there was a mean change in reported frequency of consumption of dietary fat sources of –0.39 times/day (*P* < .001) and an increase of 0.73 servings of fruits/vegetables/day (*P* < .001) (Table 5). When all participants are examined, including nonrespondents to the evaluation questionnaire (assigned a change score of zero plus a random variable), there is still a significant change in dietary practice: a decrease in frequency of consumption of dietary fat of –0.22 times/day (*P* = .013) and an increase of 0.37 servings of fruits/vegetables/day (*P* = .002).

Again, we examined the internal consistency of relationships between reported dietary change and the extent of participation in the program. Extent of participation was captured by the program and was not biased by self-report. Change in fruit and vegetable consumption was significantly associated with number of weeks the participant had interacted with the program (*P* = .01) (data not shown). This was true despite the fact that nonrespondents to the evaluation were assigned a change score of zero plus a random variate. There was no significant association between change in consumption of fat sources and number of weeks the participant had interacted with the program.

## Discussion 

This study demonstrates that it is feasible to deliver an e-mailed nutrition intervention program in a corporate worksite setting and suggests that the WIN program can achieve significant improvements in stage of dietary change and in dietary behavior. Statistically significant improvements in both fruit and vegetable intake and reductions in fat intake were seen, even when nonrespondents to the evaluation questionnaire were assigned a change score of zero. However, because of the lack of a randomized design and the 56% response rate, more definite conclusions must await further research with this intervention program.

As noted, individual tailoring to the participant's lifestyle was an important feature of the WIN program. Extensive literature supports the value of tailoring to increase the effectiveness of interventions (5,19-21). Campbell et al have shown that tailoring enhances the effectiveness of simple messages in improving dietary behavior (22). For example, four months after a single mailed intervention, those receiving tailored messages more often recalled receiving the dietary information, were more likely to have read all of it, and reported significantly less total fat and saturated fat intake than those receiving more traditional, untailored messages. Customizing messages to individual characteristics was a key feature of WIN, although the tailoring focused on practical aspects of the individual's life rather than on Stage of Change and self-efficacy.

The primary limitation to confidence in the results of this study is that the study is not a randomized controlled trial. The purpose of the study was to test, in a real-world situation, the feasibility of the delivery method and the participation of and acceptability to a company and its employees. Application of a randomized design would not have served this purpose, nor did the available funding and time frame permit it. However, the dose-response relationship between apparent effectiveness and extent of participation provides an internal consistency that suggests a real effect. Moreover, it is notable that many of the same behavioral principles applied in WIN were applied to the development of the *Little by Little* CD-ROM, whose effectiveness in improving dietary behavior was demonstrated in a randomized placebo-controlled trial (23).

The 56% response rate to the follow-up questionnaire is also a limitation. However, this response rate is above the cut point for minimal acceptable response rate as defined by Ammerman et al and Pignone et al (5,15) for the Preventive Services Task Force. In addition, we attempted to overcome the potential for selection bias by setting nonresponders to zero change for some analyses and by examining the internal relationship between effect and extent of interaction with the e-mails, a measure unbiased by self-reporting.

Finally, the diet change scores are based on self-reports. It would have been desirable to obtain blood levels or a more rigorous self-report method such as detailed dietitian-administered 24-hour dietary recalls. We hope to be able to do this in a randomized controlled trial.

We believe that the WIN program may have some relevance to clinical practice. While health care professionals are encouraged to consider behavioral counseling of their patients to promote a healthy diet (2), time is a constraint (24). However, the Preventive Services Task Force describes the following as "promising for the general population of adult patients in primary care settings": "Lower-intensity interventions that involve five minutes or less of primary care provider counseling supplemented by patient self-help materials, telephone counseling, *or other interactive health communications*" (emphasis added) (2). The program described here could serve this purpose.

WIN is particularly appropriate for population-wide health promotion. As of 2001, 56.5% of U.S. households had a personal computer, and two thirds of Americans used a computer at some location, including at work, a public library, a community center, or someone else's home (25). Internet use has been growing at a rate of 20% per year. As of mid-2003, it was estimated that there were 126 million unique Internet users in the United States (63% of all adult Americans) (26). While there are ethnic and income differences, the information gap is narrowing. Even among persons in the lowest income category (<$15,000/year), approximately 25% were computer users in 2001, and that proportion is growing at a rate of 25% a year (25). Approximately 44% of Hispanics and 46% of African Americans are regularly online (27).

E-mail, in particular, is becoming a part of the fabric of American life. As of December 2002, 102 million Americans were e-mail users (87% of online African Americans, and 93% of online whites) (26). The particular advantage of the e-mail system used in WIN is that it does not rely on participants to initiate information-seeking behavior. That is, information comes to the user, rather than the user having to go and look for it. Only 7% of persons with Internet access actively look for health and medical information on a typical day, whereas 52% of Internet users send and receive e-mail on a typical day (27). 

More directly, this pilot study of the WIN program is relevant to worksite health promotion. Worksite interventions can be effective in changing behaviors and may reduce health care costs (28,29). However, their complexity and cost make them infeasible for many businesses. An e-mail–based program can make health promotion accessible to many, while retaining the scientific basis critical to behavior change.

The present study demonstrates the feasibility of delivering an e-mail–based tailored dietary intervention in a worksite and provides evidence that such an intervention may produce improvements in both Stage of Change and in dietary intake of fruits and vegetables and of fat. This intervention, with its emphasis on dietary assessment and tailoring to the participant's lifestyle, could bring widespread dietary screening, counseling, and effective behavior change to large numbers of Americans at relatively low cost.

The WIN program may be obtained by contacting Block Dietary Systems, www.nutritionquest.com.

## Figures and Tables

**Table 1:** Principles and Program Components, E-mailed Worksite Internet Nutrition (WIN) Program, Northern California, 2001

**Program Components**	**Principles**						

	**Relevance to Learner**	**Tailoring to Individual**	**Flexibility and Individual Choice**	**Skill Facilitation**	**Commitment and Goal Setting**	**Reminders and Reinforcement**	**Multiple Strategies and Channels**
Baseline dietary screening questionnaire and feedback	Identified participant’s problem areas	NA^a^	Permitted participants to choose dietary area that most interested them	NA	Need for commitment is clearer when participant’s own problem areas are clear	NA	NA
Baseline lifestyle questionnaire	Permitted tailoring to participant’s life situation	Key element in tailoring of tips and goals	NA	NA	NA	NA	NA
Family members were encouraged to join	NA	NA	NA	NA	NA	Family members could support each other	Social support
Weekly “Did you know?”	NA	NA	NA	NA	NA	Maintained interest	Stimulated discussion with family, coworkers
Weekly *Health Notes*	Health issues related to both sexes, all age groups were highlighted	NA	NA	Some provided info on topics such as “What is a serving?”	NA	Maintained interest	Made other health news more salient
Weekly Tips and Ideas (Table 2)	Only tips relevant to participant’s life situation were provided	Tailored to individual’s chosen dietary emphasis and lifestyle path	NA	Provided tips on simple skills	NA	NA	Some involved things family members could do
Weekly goal setting	Only goals relevant to participant’s life situation were suggested	Suggested goals were tailored to individual’s life situation	Participant could choose one or two of the suggested goals or choose to continue with one selected in a prior week	Goals included developing skills necessary to achieve the dietary goal	Participant committed to goals in simple declarative statements (e.g., “I will put a bowl of fruit on the kitchen table.”)	Each week, participants were asked about their success and were congratulated or encouraged	NA

a NA indicates not applicable.

**Table 2:** Table 2. Lifestyle Paths^a^ and Examples of Individualized Tips and Goals^b^, E-mailed Worksite Internet Nutrition (WIN) Program, Northern California, 2001

**Lifestyle Path**	**Sample Tip**	**Sample Small-Step Goal**
Most dinners at home, participant cooks, no kids at home	Eat different kinds of vegetables and fruits each day. Each vegetable or fruit has its own unique package of disease-preventing nutrients. Variety is the spice of life!	I will try to eat one new fruit and one new vegetable this week (different from what I usually eat).
Most dinners at home, participant doesn’t cook	If you aren’t fond of the vegetables served at dinner and you often pass on eating them, include a piece of fruit with dinner instead.	I will include fruit for dessert at dinner every other day this week.
Most dinners at home, participant cooks, kids at home	It’s hard for you to eat low fat when your family doesn’t wantto. Here are some ideas. When you go shopping, let your kids pick out one new low-fat food to try. They may find they like some of them — like graham crackers, angel food cake, nonfat yogurt, low-fat saltines, soft pretzels.	I will talk to everyone in the family to find out what low-fat foods each member might like to include in the meal and snack menus.
Frequent dinners out, participant cooks	Get your vegetables in when you eat pizza — go for extra sauce, bell peppers, onions, mushrooms, spinach, artichoke hearts or whatever appeals to you.	This week, I will include a serving of vegetables on the side whenever I have a fast food meal.
Frequent dinners out, participant doesn’t cook	Instead of a biscuit sandwich for lunch or breakfast, choose any other kind of bread or roll. Pass on the butter or mayonnaise.	I will substitute a French or sourdough roll for a biscuit on a take-out sandwich this week.

a Two additional lifestyle paths were defined: persons whose dinners are equally divided between home and in restaurants, with and without children at home. However, 96% of participants fell into one of the five paths mentioned above.

b For each of these lifestyle paths, there was a separate set of tips and goals for persons working on fat intake and fruit and vegetable intake.

**Table 3:** Participant Satisfaction Among Respondents to the Evaluation Questionnaire, E-mailed Worksite Internet Nutrition (WIN) Program, Northern California, 2001

**Question**	**%**

**Any technical difficulties?**	

No	94
Yes	6

**Nutrition feedback and tips helpful?**	

No	7
Yes	93

**Online discussion board**	

Helpful	19
Never visited	79
Not helpful	2

**Would you recommend this program?^a^**	

No	11
Yes	83
No response	6

**How much time it took**	

About right	64
Would prefer shorter	25
No response	11

**Why didn’t participate more?**	

Worried about confidentiality	0
Too busy, would do later	23
Too much time required	11
Other reason^b^	15
No response	51

**Talked with someone at work about improving diet?**	

No	68
Yes	30
No response	2

**Talked with someone at home about improving diet?**	

No	51
Yes	47
No response	2

**Read at least half the e-mails?**	

No	17
Yes	83

**Learn anything about your eating?^c^**	

No	28
Yes	70
No response	2

**What dietary factor did you work on?**	

Both fruits/vegetables and fat	55
Fruits/vegetables only	26
Fat only	15
No response	4

a Among the 50% who were “budget-conscious,” 100% would recommend the program. Among the 50% who were not already “Health conscious, already eat a healthful diet,” 100% would recommend the program. Among males, 100% would recommend the program.

b Other reasons for not participating more in the program included illness, being out of the office a lot, program not targeted to nutrients respondent was interested in, or already eat a low-fat, high fruit and vegetable diet.

c Among the 53% who were not “Health conscious, already eat a healthful diet” at baseline, 80% said they learned something new.

**Table 4:** Improvement in Stage of Change, E-mailed Worksite Internet Nutrition (WIN) Program, Northern California, 2001

	**Amount of Change^a^> **	**Percentage With Upward Movement**	***P* b**

**Stage of Change: fat**			

**Evaluation respondents**			

All (n = 47)	0.36	65	<.001
Those not already at top (n = 26)^c^	0.65		<.001
		
**Original program participants**			
		
All (n = 84)^d^	0.20		<.001
Those not already at top (n = 42)	0.40		<.001

**Stage of Change: fruits and vegetables**			

**Evaluation respondents**			

All (n = 47)	0.57	74	<.001
Those not already at top (n = 23)^c^	1.17		<.001
		
**Original program participants**			
		
All (n = 84)^d^	0.32		<.001
Those not already at top (n = 39)	0.43		<.001

a For Stage of Change, one unit represents movement of one step up.

b Is mean change significantly different from zero? (Determined by *t*-test.)

c Includes only respondents who were not already in Action/Maintenance at baseline.

d Original participants who did not complete the evaluation questionnaire were assigned a follow-up Stage of Change identical to their baseline Stage. This was also true for two persons in the evaluation group whose Stage of Change was missing at the evaluation.

**Table 5:** Change in Dietary Behavior, E-mailed Worksite Internet Nutrition (WIN) Program, Northern California, 2001

	**Amount of Change^a^**	***P* b**

**Fat sources: amount of change**		

Evaluation respondents (n = 47)	-0.39	<.001
All original program participants (n = 84)^c^	-0.22	.01

**Fruits and vegetables: amount of change**		

Evaluation respondents (n = 47)	0.73	<.001
All original program participants (n = 84)^c^	0.37	.002

a Change in times per day consumption: for fat sources, consumption of 17 foods (hamburgers, beef, fried chicken, hot dogs, lunch meats, bacon/sausage, salad dressing, butter/margarine on bread/vegetables, butter/margarine/oil in cooking, eggs, pizza, cheese, whole milk, French fries, chips, doughnuts/pastries, ice cream). For fruit and vegetable sources, times per day consumption of seven foods (fruit, fruit juice, vegetable juice, salad, potatoes [not fried], other vegetables, vegetable soup). See Methods for scoring.

b Is mean change significantly different from zero? (Determined by *t*-test.)

c Original participants who did not complete the evaluation questionnaire were assigned a follow-up score identical to their baseline score, plus a random variate with mean = zero and SD = the SD of evaluation respondents.
